# A two-centre study investigating the association between brain natriuretic peptides & outcomes in continuous flow left ventricular assist device recipients

**DOI:** 10.1186/s13019-025-03422-w

**Published:** 2025-04-16

**Authors:** Maria Lambadaris, Farid Foroutan, Julie KK Vishram-Nielsen, Marie Sophie S. Knudsen, Filio Billia, Vivek Rao, Finn Gustafsson, Ana C Alba

**Affiliations:** 1 Heart Failure and Transplantation Program, Toronto General Hospital, University Health Network, Toronto, ON Canada; 2 Department of Cardiology, Rigshospitalet, University Hospital of Copenhagen, Copenhagen, Denmark; 3 University of Toronto, 500 University Ave. #602, Toronto, ON M5G 1V7 Canada

**Keywords:** Heart failure, Left ventricular assist device, Heart transplantation, Brain natriuretic peptides

## Abstract

**Background:**

Natriuretic peptides are useful prognostic markers in patients with heart failure and this study aims to evaluate the association between N-terminal pro-brain natriuretic peptide and outcomes in patients with a continuous-flow left ventricular assist device.

**Methods:**

Adult patients with a continuous-flow left ventricular assist device as bridge to transplant or destination therapy and discharged from hospital were included in the study. Multi-variable Cox regression models with natriuretic peptide as a time-dependent covariate modelling multiple measures per patient, and cubic spline analysis were used to evaluate the association between natriuretic peptides and patient outcomes, taking median N-terminal pro-brain natriuretic peptide of 244 pmol/L as the reference value. Heart transplant was considered a competing event for mortality analysis, while death was considered a competing event for transplantation analysis. A multivariable cause-specific Cox regression model evaluated the relationship between natriuretic peptides and heart failure hospitalizations while considering heart failure hospitalizations as recurring events and adjusting for competing risks of death and transplant.

**Results:**

This retrospective cohort study included 161 adults. During a median follow-up of 4.7 years, 49 patients died, 79 received a heart transplant and 66 were hospitalized for heart failure. In comparison to the median N-terminal pro-brain natriuretic peptide level, patients exceeding 350 pmol/L had a 19% increased mortality (HR 1.19, 95%CI 1.01–1.15) while patients exceeding 330 pmol/L had a 16% higher risk of hospitalization (HR 1.16, 95%CI 1.01–1.12).

**Conclusion:**

In patients with a continuous-flow left ventricular assist device, serial assessments of natriuretic peptides may help provide personalized patient care.

**Supplementary Information:**

The online version contains supplementary material available at 10.1186/s13019-025-03422-w.

## Graphical Abstract

This multi-institutional study aimed to evaluate the association between NTproBNP and outcomes in patients with a CF-LVAD as a bridge to HTx or DT. Mortality and risk of hospitalization increased with increasing values of NTproBNP and therefore, NTproBNP may help to guide personalized care and if NTproBNP is significantly elevated, it may warrant intensified care.

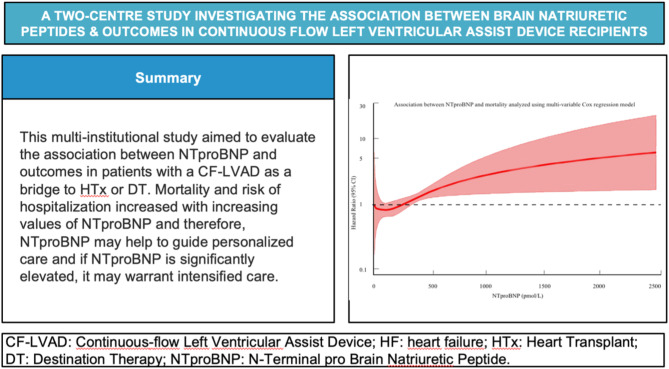

## Introduction

Patients with advanced heart failure (HF) can benefit from left ventricular assist device (LVAD) therapy, which improves survival and quality of life. While Brain Natriuretic Peptide (BNP) and N-terminal proBNP (NTproBNP) have been extensively studied in ambulatory HF patients, and proven useful prognostic markers, there is scarce and inconsistent evidence on their prognostic value in LVAD patients.

The available literature which has examined the association of natriuretic peptides and outcomes in LVAD patients include both pulsatile and continuous-flow LVADs, have short follow-up times, and evaluate BNP at specific points in time or compare relative changes in BNP to baseline measurements [1–3]. For example, in a study of 83 patients with a mean follow-up time of 2 years, BNP less than 322 pg/ml at 60 days post-LVAD implant was associated with 92% survival compared with 70.5% in those where BNP exceeded 322 pg/mL^1^. However, this study incorporated both continuous-flow (CF) and pulsatile-flow LVADs and only evaluated BNP at a specific time point. In another single centre study, serial BNP measurements were done in 136 outpatients post-HeartMate II LVAD as a bridge to transplantation [2]. They found that an absolute BNP value greater than 609 ng/L was significantly associated with a severe event requiring hospitalization (i.e. infection, heart failure exacerbation with or without pump dysfunction). When examining absolute BNP measures and changes from baseline BNP for each patient, there was no association between BNP and mortality or hospitalization at 180 days following device implantation [3]. Conversely, Mehra et al. demonstrated that the primary reason for rehospitalization and death in patients with CF-LVAD support is due to a heart failure syndrome [4].

Due to these controversies, we evaluated the association between serial measurements of NTproBNP and long-term outcomes, including mortality, heart transplantation (HTx), and HF hospitalizations, in patients discharged from hospital who underwent CF-LVAD as a bridge to HTx or destination therapy (DT) in this multi-institutional larger study.

## Materials and methods

### Study design and patient population

This is a retrospective cohort two-centre study (Toronto General Hospital in Canada and Rigshospitalet in Denmark) of consecutive adults (above 18 years old) with a CF-LVAD for DT or bridge to HTx and discharged from index hospital admission between January 1, 2006 and December 31st, 2020. Patients that had a planned LVAD insertion but then required an RVAD intra-operatively are included, as are patients with a pre-existing LVAD (i.e., L-centrimag) who received a durable LVAD device.

Patients that did not have any BNP or NTproBNP values post-discharge from the index hospital admission were excluded. Patients that died in the index LVAD implantation hospitalization were excluded. Patients only supported with short term devices (e.g., RVAD or BIVAD or percutaneous LVAD) without subsequent long-term LVAD were excluded. Patients with pre-existing RVAD before LVAD were excluded. Patients with percutaneous LVAD as a bridge to recovery were also excluded.

### Ethical statement

This work was approved by the respective ethics board at each hospital site (#17-6078, January 2018).

### Clinical variables collected

Demographic and clinical information was extracted from the patient records (EPR, Epic). Baseline characteristics and laboratory values prior to LVAD implant were collected and included: patient age at the time of CF-LVAD implant, sex, race, height, weight, history of diabetes, hypertension, COPD, smoking, peripheral vascular disease, renal disease, dialysis, type of cardiomyopathy, INTERMACS classification, type of CF-LVAD device, hemoglobin and creatinine. Variables post-LVAD and pre-transplant included date of hospitalization, reason for hospitalization, last follow-up, and multiple measures of natriuretic peptides (e.g. BNP or NTproBNP) after discharge (from the index LVAD implantation hospitalization). BNP and NTproBNP were measured in the centralized laboratory of each institution and collected at a minimum of one day post-discharge with no upper limit. Goal-directed medical therapy reflects the medications that the patients received at the time of discharge from the hospitalization where the LVAD was implanted. During data collection, each hospital admission was carefully reviewed and classified according to the main reason for admission. Heart failure was the main course in patients presenting with increased exertional dyspnea, orthopnea or paroxysmal nocturnal dyspnea, bilateral peripheral edema and increased jugular vein pressure. Due to the interconnection between cardiac and renal function, patients with worsening renal function presenting with the aforementioned heart failure symptoms or signs were coded as heart failure. For patients with events, the follow-up ended at the time of death and for patients without events, the follow up ended at last observation in the electronic patient record before the final data collection date of December 31st, 2020.

### Natriuretic peptide measurement and conversion

BNP was measured during routine out-patient follow-up and within the first 24 hours that a patient presented to the hospital for an event. Specifically, at the Toronto General Hospital, patients are followed-up after discharge every 2 weeks for 3 months, and then every month for 6 months, and then every 2 months until one year. Thereafter, patients are seen in clinic every 3–6 months. Most patients have their BNP levels measured at the time of the clinic visit and within 24 hours of hospital admission. Biomarkers are also measured prior to ramp study and within weeks after any speed or therapy modifications.

At the Rigshopitalet, LVAD-VAD recipients are followed-up as follows: every day for the first 14 days post-implant, then at day 21 and 28. After this the patients are seen on a monthly or bimonthly basis if on transplant waitlist, every three months if DT. NTproBNP is taken every third month.

The Toronto General Hospital (TGH) measures natriuretic peptides using BNP (pg/mL) while Rigshospitalet (RH) uses NTproBNP (pmol/L**)**. We converted BNP to NTproBNP based on the formula published in the study by Masson et al.: NTproBNP = 3.48 × BNP − 19 (correlation coefficient r^2^ = 0.94) [5].

### Post-LVAD heart failure care

At both centers (TGH and RH), patients received standard heart failure care post-LVAD insertion. At TGH, patients were prescribed maximumtolerated guideline directed medical therapy (GDMT) such as, beta-blockers, mineralocorticoid antagonists (MRA), angiotensin converting enzyme inhibitors (ACEi), angiotensin receptor blockers (ARB), hydralazine, isosorbide dinitrate (ISDN) and sildenafil. Of note, there is decreased use of angiotensin receptor blocker neprilysin inhibitors (ARNI) at the TGH due to patients having undergone a recent hospitalization and high risk surgery. Furthermore, patients that are bridged to HTx do not receive ARNI at TGH to reduce risk of vasoplegia at the time of HTx. At the RH, patients received maximally tolerated beta-blocker, MRA, and ACEi or ARB.

At both institutions, all patients underwent an echo-guided ramp study to adjust LVAD speeds to ensure adequate left ventricular unloading (e.g. reduce left ventricular size and mitral regurgitation) and permit aortic valve opening intermittently. The latter occurs prior to patient discharge (TGH), 3 months post-discharge (RH) and then annually (TGH and RH), thereafter.

### Classification of outcomes

Outcomes observed after discharge from the index LVAD implant hospitalization were classified into the following groups:

1. Acute decompensated HF hospitalization– defined as any hospitalization that was documented in the patient chart as acute decompensated heart failure, new or worsening shortness of breath, or volume overload.
2. All-cause mortality.
3. Cardiac transplantation.
4. Composite end-point of first hospitalization for acute decompensated heart failure and mortality.

Admissions for implantation of ICD, AICD pulse generator change, and heart transplantation were excluded. In patients undergoing heart transplant or LVAD explant, follow-up was censored at the time of intervention as the patient was alive.

### Statistical analysis

Categorical variables were presented as proportions and continuous variables were represented as the median and 25th and 75th percentiles. We compared characteristics among the two centres using the Student’s t-test and Kruskal Wallis.

To evaluate the association between NTproBNP and mortality, we used a multivariable Cox regression model, with NTproBNP entered as a time-dependent covariate modelling multiple measures per patient, and using cubic spline, with heart transplantation as the competing event. Inputting NTproBNP as a time-dependent covariate allowed us to capture changes in this measure throughout the follow-up time of patients since, NTproBNP changes over time. The change in NTproBNP and its impact on the hazard function is effectively captured by considering NTproBNP as a time-dependent covariate. We used a cause-specific hazard approach to account for multiple competing events specifically, death and heart transplantation. This method allowed us to separately estimate the hazard for each competing event, ensuring that we capture the distinct risks associated with death and heart transplantation while considering that experiencing one event precludes the occurrence of the other. For the cubic spline analysis, the reference was set to median measurement of BNP for the cohort, 244pmol/L. Multivariable analyses were adjusted for a priori selected limited number of clinically important factors to prevent overfitting; these included age, sex, diabetes, and ischemic cardiomyopathy as the cause of HF. To account for other potential confounders, we also included a second multivariable analysis which adjusted for creatinine, dialysis, BMI, centre and intention of LVAD therapy. The use of cubic splines ensures that we are not assuming a linear relationship between BNP and the natural logarithm of the hazard function in the model. Cubic splines or restricted cubic splines allow for flexible modeling of the relationship between a continuous covariate and the hazard function by fitting a series of smooth, connected curves. This avoids assuming a strict linear relationship and can better capture complex, non-linear effects, especially for a covariate like BNP for which we think there is a threshold effect. To evaluate the association between NTproBNP and transplant, we used a multivariable Cox regression model, with NTproBNP as a time-dependent covariate modelling multiple measures per patient, and using cubic spline, with death as the competing event. The reference for the cubic spline analysis was set to the median measurement of BNP for the cohort, 244 pmol/L. Lastly, we analyzed the association between NTproBNP and HF hospitalizations. Hospitalization due to HF was considered as a recurring event. NTproBNP levels, our primary predictor, was transformed on the natural log scale (ln_bnp) and analyzed using a Cox proportional hazards model with a counting process approach to accommodate recurrent events. We applied restricted cubic splines with 3 knots to model ln_bnp, accounting for its nonlinear relationship with the risk of hospitalization. Clustering by patient was considered in the analysis to address intra-cluster correlations, and robust standard errors were estimated to enhance the model’s validity.

### Data availability

Data is available upon request.

## Results

### Baseline characteristics

During the study period, a total of 185 patients received a CF-LVAD at the Toronto General Hospital (Supplementary Figure S1). Of these, 80 were excluded (32 patients died after LVAD implant during the index hospitalization, 41 patients did not have BNP values, 5 patients received a heart transplant in the index hospitalization, 1 person had LVAD recovery and explant and 1 person was lost to follow-up). A total of 104 patients received a CF-LVAD at the Rigshospitalet. Of these, 48 were excluded (47 did not have NTproBNP values and 1 person died in the index hospitalization). Between the two centres, a total of 161 patients (105 from Toronto General Hospital, 56 from Rigshospitalet) were included in the analysis (Table 1). The median age of the cohort was 54 years [46.2 and 63.8 years], 16% were female, and 44% had ischemic cardiomyopathy. With respect to device therapy, 37 (23%) patients were supported with a Heartware HVAD (HeartWare, Framingham, MA) device, 77 (48%) patients with HeartMate II (Abott Laboratories, Abbott Park, IL), 46 (29%) patients with HeartMate III (Abott Laboratories) and 1 (1%) patient with a Duraheart device (Terumo Heart Inc, Ann Arbor, MI). The baseline characteristics were mostly similar between the two centres (Supplementary Tables 1, 2). We did not find any significant association between medications at discharge and patient outcomes (Supplementary Table 3).

**Table 1:** Baseline characteristics

	Overall (*N* = 161)
**Age (years)**	53.9 [46.2, 63.8]
**Gender**	
Male	136 (84.5%)
Female	25 (15.5%)
**Race**	
Caucasian/white	126 (78.3%)
Black	9 (5.6%)
Other	1 (0.6%)
**Body Mass Index (kg/m** ^**2**^ **)**	26.5 [23.1, 29.2]
**Ischemic Cardiomyopathy**	70 (43.5%)
**Diabetes**	57 (35.4%)
**Smoking**	
Never smoked	88 (54.7%)
Currently smoking (within last year)	33 (20.5%)
Past smoker	36 (22.4%)
**Atrial Fibrillation**	72 (44.7%)
**COPD**	12 (7.5%)
**Hypertension**	68 (42.9%)
**Peripheral Vascular Disease**	12 (7.5%)
**Chronic Renal Failure**	34 (21.1%)
**Dialysis**	13 (8.1%)
**INTERMACS**	
1–2	23 (14.3%)
3–4	115 (71.4%)
5–7	17 (10.6%)
**Hemoglobin (g/L)**	11.9 [9.8, 13.3]
**Creatinine (µmol/L)**	114.0 [90.1, 151.9]
**LVAD Type**	
HeartMate II	77 (47.8%)
HeartMate III	46 (28.6%)
HeartWare	37 (23.0%)
Other	1 (0.6%)
**Goal Directed Medical Therapy**	
Beta-Blocker	63 (39.1%)
Angiotensin Converting Enzyme Inhibitor	50 (31.1%)
Angiotensin Receptor Blocker	6 (3.7%)
Angiotensin Receptor Blocker/Neprilysin Inhibitor	0 (0%)
Mineralocorticoid Receptor Antagonist	85 (52.8%)
Sodium/glucose cotransporter 2 Inhibitor	0 (0%)
Hydralazine	31 (19.3%)
Isosorbide Dinitrate	9 (5.6%)
Sildenafil	33 (20.5%)
Mean Daily Furosemide Dose (mg)	86.3 [40,160]

### Association between NTproBNP and mortality

**Fig. 1:**
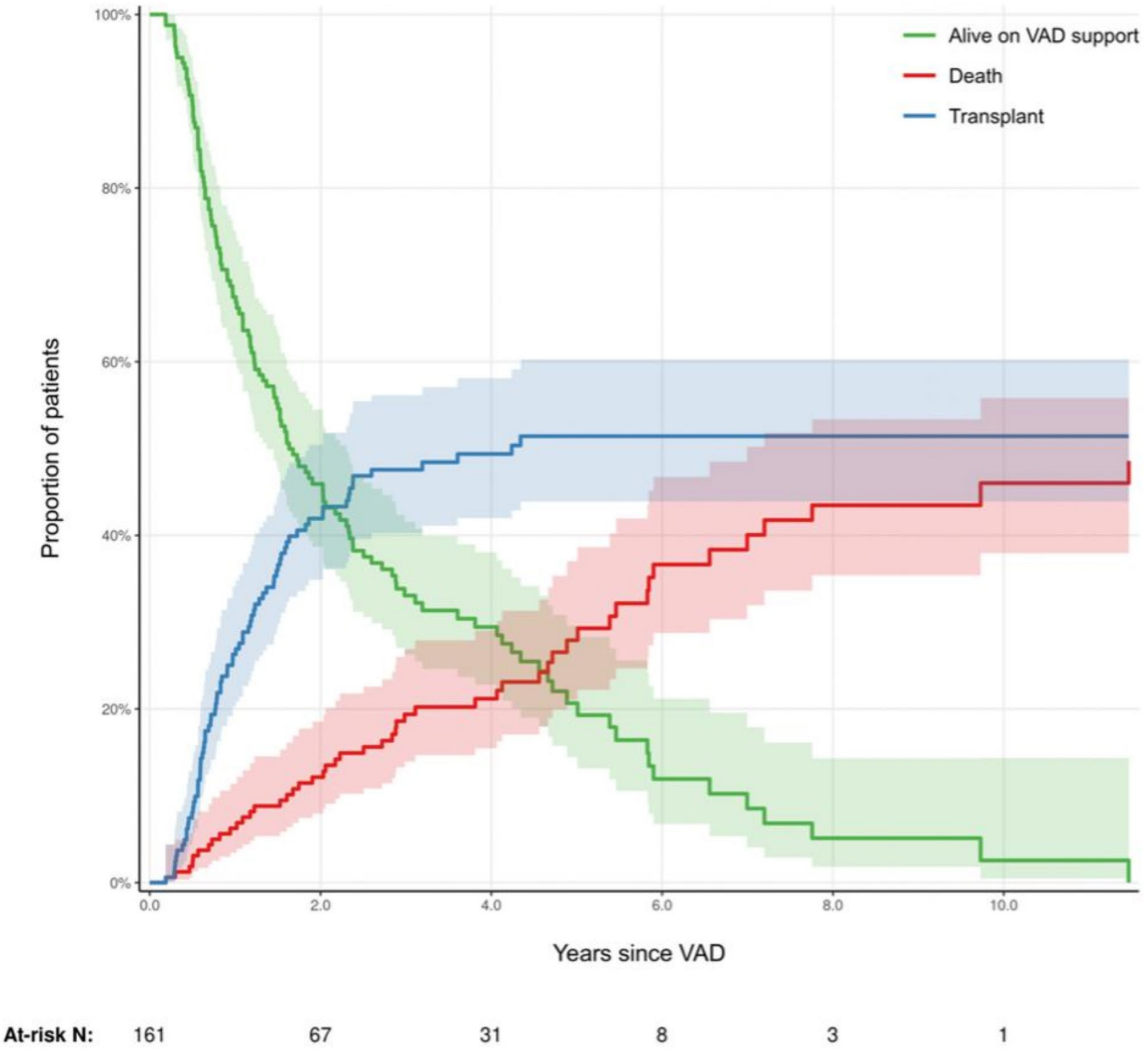
Time to event analysis The three possible event states for patients with continuous-flow left ventricular assist devices (CF-LVAD). The green curve depicts freedom from death or transplant (alive on LVAD support). The blue curve represents the cumulative incidence of transplant, adjusted for competing risk of death. The red curve represents the cumulative incidence of death, adjusted for competing risk of transplant

**Fig. 2:**
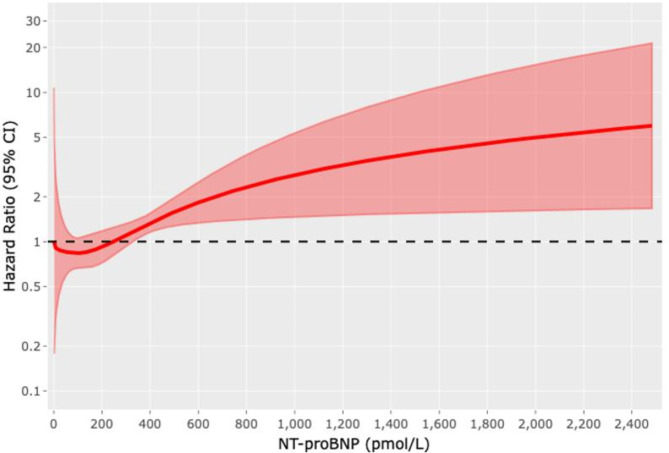
Multivariable cox regression model for mortality Association between NTproBNP and mortality analyzed using multi-variable Cox regression model. NTproBNP was entered as a time-dependent covariate modeling multiple measures per patient and using cubic spline, with heart transplantation as the competing event. The reference for the cubic spline analysis was set to median measurement of NTproBNP for the cohort, 244 pmol/L. The multivariable analysis was adjusted for age, gender, diabetes, and ischemic cardiomyopathy as the etiology of heart failure.

During a median follow-up time of 4.7 years (3.8–5.8 years), there were 49 deaths (32 at TGH and 17 at RH), 79 transplants (54 at TGH and 25 at RH) and a total of 1,813 BNP measures. There was a median number of 8 (25-75th percentile, 2–19) measurements of NTproBNP per patient at the TGH and 7 (25-75th percentile 2–12) at the RH. At 6 months post-LVAD implantation (Table 2), 90% [85.6%, 94.8%] were alive on pump, 2% [0.6%, 5.7%] had died and 8% [4.8%, 13.6%] underwent successful cardiac transplant. Figure 1 demonstrates the three possible event states for patients on VAD support and shows that a patient with an LVAD is more likely to receive a heart transplant than they are to die. By multi-variable analyses (Fig. 2, Supplementary Table 4), increasing NTproBNP levels were associated with increased mortality. In comparison to the median NTproBNP of 244 pmol/L, patients with a NTproBNP of 350 pmol/L had a mortality 19% (HR 1.19, 95%CI 1.01–1.15), those with a NTproBNP of 500 pmol/L had a mortality of 48% (HR 1.48, 95% CI 1.08–1.30, and those with NTproBNP as high as 1000 pmol/L had 2.4 fold increased mortality (HR 2.40, 95% CI1.13–1.91).

**Table 2:** Time to event Analysis– Frequency of events

Years	Alive on Support	Death	Transplant
0	100.0% [0.0%, 0.0%]	0.0% [0.0%, 0.0%]	0.0% [0.0%, 0.0%]
0.5	90.1% [85.6%, 94.8%]	1.9% [0.6%, 5.7%]	8.1% [4.8%, 13.6%]
1	67.5% [60.6%, 75.1%]	6.3% [3.4%, 11.4%]	26.3% [20.3%, 34.1%]
2	45.9% [38.7%, 54.5%]	12.1% [8.0%, 18.5%]	41.9% [34.9%, 50.4%]
4	29.4% [22.8%, 38.0%]	21.2% [15.5%, 29.0%]	49.4% [42.0%, 58.1%]
6	11.9% [6.7%, 21.2%]	36.6% [28.7%, 46.7%]	51.4% [43.9%, 60.3%]
8	5.1% [1.8%, 14.3%]	43.5% [35.4%, 53.4%]	51.4% [43.9%, 60.3%]
10	2.6% [0.5%, 14.4%]	46.0% [37.9%, 55.8%]	51.4% [43.9%, 60.3%]

### Association between NTproBNP and probability of heart transplant

**Fig. 3:**
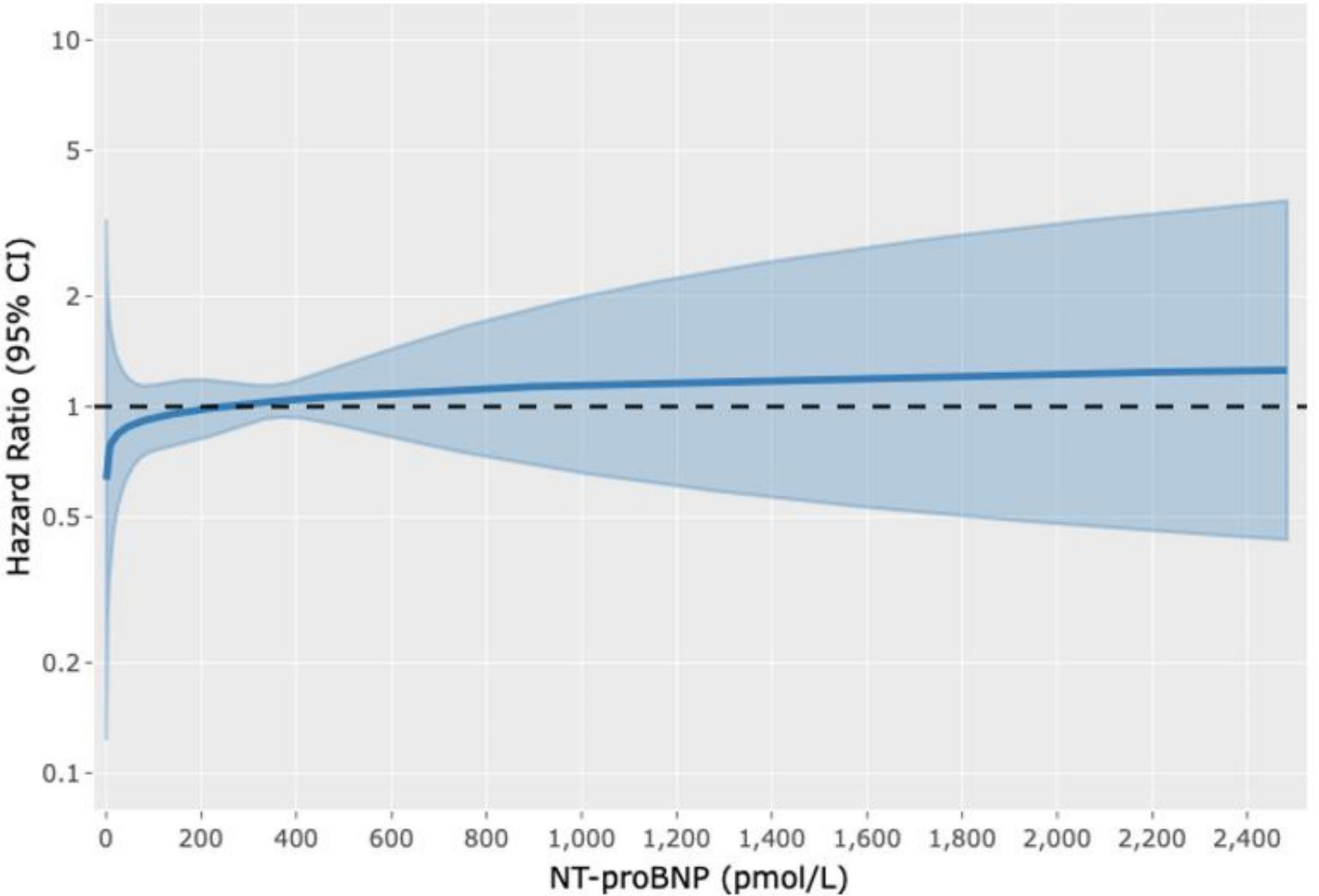
Multivariable cox regression model for heart transplantation Association between NTproBNP and heart transplant analyzed using multivariable Cox regression model. NTproBNP was entered as a time-dependent covariate modeling multiple measures per patient and using cubic spline, with death as the competing event. The multivariable analysis was adjusted for age, gender, diabetes, and ischemic cardiomyopathy as the etiology of heart failure.

By multivariable and univariable analyses, NTproBNP levels were not associated with probability of HTx after adjusting for the competing risk of death (Fig. 3 and Supplementary Figure S2, respectively; Supplementary Table 5).

### Association between NTproBNP and hospitalization

**Fig. 4:**
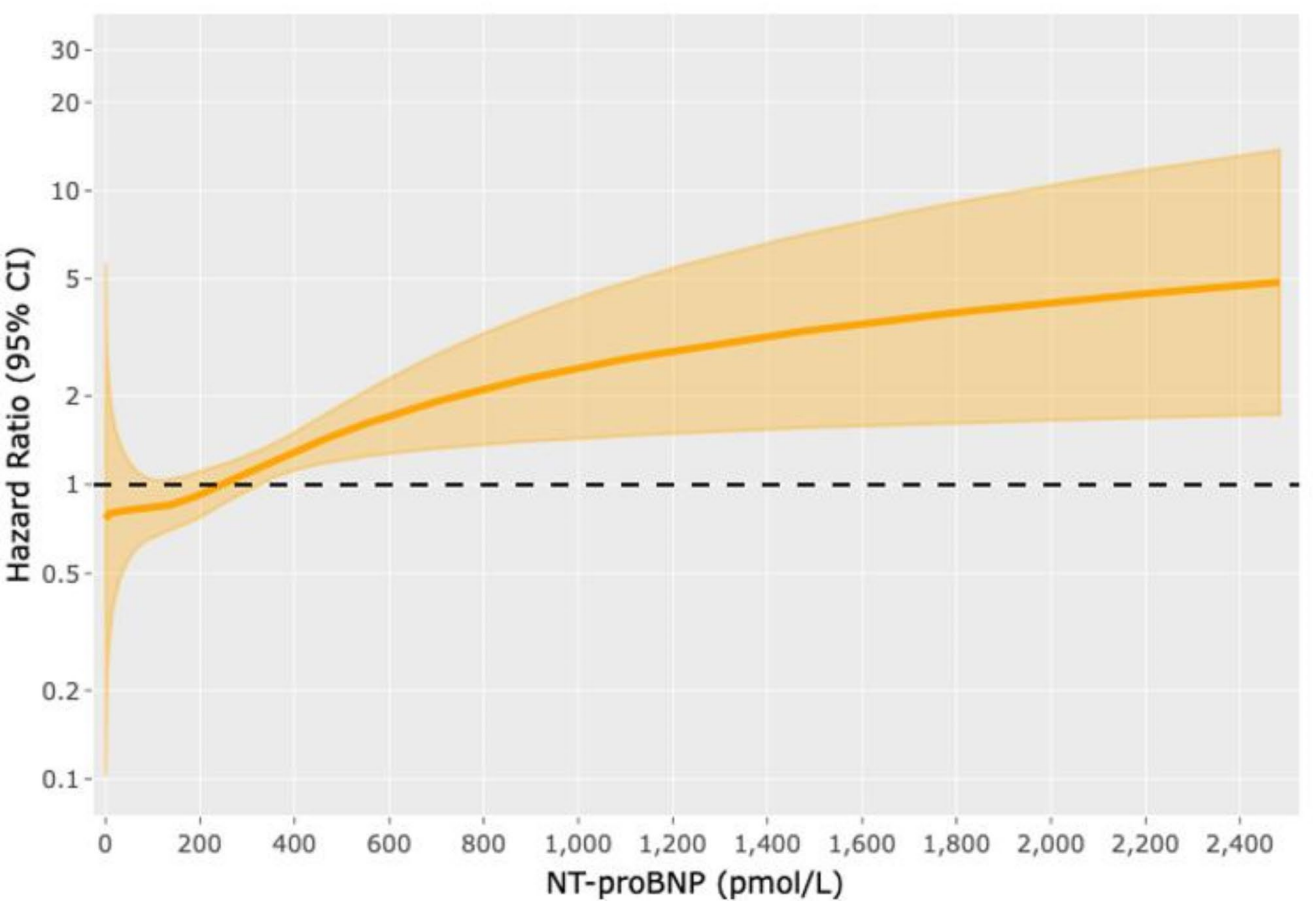
Multivariable cause-specific cox regression model for heart failure hospitalization Association between NTproBNP and heart failure (HF) hospitalization was analyzed using a multivariable cause-specific cox regression model which was adjusted for competing risk of death, transplant, and non-HF hospitalization. HF hospitalizations were analyzed as recurrent events. The multivariable analysis was adjusted for age, gender, diabetes, and ischemic cardiomyopathy as the etiology of heart failure.

During follow up, we observed 66 hospitalizations related to a HF exacerbation. Multivariable analysis (Fig. 4, Supplementary Table 6) demonstrated increased risk of HF hospitalization with increasing values of NTproBNP when compared to the median NTproBNP of the cohort, 244 pmol/L. Specifically, a NTproBNP of 330 pmol/L had a significant rise in HF hospitalization risk of 16% (HR 1.16, 95% CI 1.01–1.12), NTproBNP of 500 pmol/L had a HF hospitalization risk of 51% (HR 1.51, 95% CI 1.09–1.31) and NTproBNP of 1000 pmol/L had a 2.5 fold increase in the risk of HF hospitalization (HR 2.51, 95% CI 1.17–1.90).

### Association between NTproBNP and outcomes when adjusting for other variables: renal dysfunction, BMI, centre, and intention of LVAD therapy

When adjusting for creatinine, dialysis and BMI, we found no significant association between NTproBNP and cardiac transplantation (Supplementary Figure S3, Supplementary Table 7). However, there was a significant association between NTproBNP and mortality when NTproBNP levels exceeded 400 pmol/L (Supplementary Figure S4, Supplementary Table 8). When adjusting for centre and intention of LVAD therapy, we found no significant association between NTproBNP and mortality or heart transplantation. (Supplementary Figs. 5, 6, Supplementary Tables 9, 10).

## Discussion

Our study demonstrated that NTproBNP can be a useful prognostic marker in patients with a CF-LVAD as bridge to HTx or DT. High NTproBNP was associated with an increased risk of mortality and risk of hospitalization due to HF exacerbation. In Canada and Denmark, HF symptoms or hospitalizations are not considered when deciding heart transplant listing priority. As demonstrated, we did not expect to find a significant association between NTproBNP and the likelihood of heart transplant.

Natriuretic peptides are synthesized in the ventricles and released into the circulation in response to ventricular expansion and volume overload. BNP is cleaved into BNP and the inactive NTproBNP peptide, and both are useful markers for the diagnosis and prognosis in congestive HF [6–8]. The effects of BNP include vasodilatation, natriuresis, and diuresis, which may improve the strain of the failing heart. Several studies in patients with LVADs have shown that levels of BNP reach a steady state within 30 to 60 days after implantation and this could be related to the recovery of native ventricular function or more frequently reduced ventricular overload and left ventricular size [9, 10]. This coincides with a small study of 36 patients with an LVAD which demonstrated that structural reverse remodeling is complete by 60 days following LVAD impantation [11].

Few studies have investigated the association between natriuretic peptides and outcomes in patients with LVADs. The evidence to date is controversial with short follow-up and cohorts that include both pulsatile and CF-LVADs. A single-centre study of patients with a CF-LVAD with a median follow-up of 10 months [2] demonstrated that BNP > 327 ng/L (or > 2,622 ng/L when converted to NTproBNP) was associated with any adverse events; however, this was in a univariable model. In other cases, they showed that BNP > 609 ng/L (equivalent to NTproBNP 5,019 ng/L) was associated with severe adverse events, BNP > 419 ng/L (equivalent to NTproBNP 3,403 ng/L) was associated with HF and BNP > 783 ng/L (equivalent to NTproBNP 6,499 ng/L) with pump thrombosis. In a different single-centre study, consisting of 83 patients with primarily pulsatile LVADs, BNP exceeding 322 ng/L (equivalent to NTproBNP 2,578 ng/L) at 60 days post-device implantation was an independent predictor of all-cause mortality [1]. A more recent single-centre study of 100 patients with CF-LVADs did not find an association between absolute BNP measurements or changes from baseline BNP and a composite outcome of all-cause death or hospitalization [3]. However, this latter study only followed patients for 6 months. In contrast, a single-centre study consisting of 98 patients with a CF-LVAD showed an exponential decrease in NTproBNP following device implant with stabilization at 60 days. Neither pre-implant nor 60-day NTproBNP measurements were associated with survival or readmission but, this study did demonstrate that a greater relative decrease in NTproBNP from pre-implant to 60 days post-implant was significantly associated with reduced risk of hospitalization [12]. Finally, a retrospective cohort study by Ali et al. followed 127 patients with CF-LVADs for a median follow-up time of 17 months and they found that for each 1000 unit increase in NTproBNP at 3 months post-discharge from CF-LVAD implantation, there was a 17% increased risk of HF hospitalization or death [13]. Although there is some evidence that natriuretic peptides have shown to be useful in prognosticating outcomes in patients with an LVAD, it should be noted that this is only within a short time frame and long-term implications cannot be inferred. Our study followed patients for more than 4 years, during which we found that NTproBNP was a clinically useful prognostic marker both in the short- and long-term follow-up in this specific patient population.

### Implications for research and clinical practice

BNP and NTproBNP are readily accessible and cost-effective tests that can be seamlessly incorporated into routine bloodwork. In our study, we found that a small increase in NTproBNP levels was associated with increased mortality (> 350 pmol/L) and HF-related hospitalizations (> 330 pmol/L). Clinicians assessing natriuretic peptides routinely in CF-LVAD out-patients can guide their care plan based on levels, and potentially mitigate unfavorable outcomes such as heart failure exacerbations, hospitalizations, or mortality. This can include up-titration of goal-directed medical therapy and optimization of LVAD settings. Several studies have demonstrated a relationship between LVAD speed adjustments (guided by ramp study) and a reduction in HF hospitalization rates, BNP levels and improvement in right ventricular function [14–16]. Our study found no association between NTproBNP levels and probability of cardiac transplant. This could be related to the inclusion of some patients who were not listed for cardiac transplantation and the lack of use of NTproBNP to guide timing for cardiac listing. Whether high natriuretic peptides levels could justify early listing for transplant of eligible patients seems a potential use, although not investigated yet.

Future research should focus on further validating the role of BNP and NTproBNP in patients with CF-LVAD. Larger studies will allow for further confounders to be incorporated into the analysis including renal function and obesity. Conversely, larger patient cohorts would enable us to investigate potential associations between natriuretic peptides and other types of cardiac related hospitalizations such as LVAD pump thrombosis, arrhythmias and non-cardiac events (i.e. sepsis) that are less frequently encountered.

### Study strengths and limitations

To our knowledge, our study is the largest multicentre study to date exploring the association between NTproBNP and outcomes in patients with contemporary CF-LVADs. Notably, our cohort had an extended follow-up time of 4.7 years, the longest duration reported in the existing literature. Furthermore, by converting BNP levels we were able to analyze both BNP and NTproBNP biomarkers which allowed us to include more patients into our analysis while also allowing our results to be applied to centres using either biomarker. Finally, in our analysis, we modelled multiple measures of NTproBNP which provides the possibility to take into account the fluctuations of NTproBNP over time, mimicking real world patient care, and provides more reliable prognostic information that could be used to guide patient care.

The current study is a retrospective cohort study and has inherent limitations. Patient outcomes were investigator reported based on complete review of the electronic chart and this is subject to bias. Due to the small sample size, we could not evaluate specific causes of death. A non-fatal event can increase the chances of having a heart failure exacerbation and coding the actual cause of death in many instances can be challenging. For example, heart failure can lead to renal dysfunction or an infection can result in heart failure. Therefore, we evaluated the association between NTproBNP and all cause death which could underestimate the association between NTproBNP and death as some patients may have died due to non-heart failure causes. The sample size also limited adjusting for all potential confounders. Other important extracardiac factors that impact NTproBNP levels in patients with an LVAD, such as renal function and obesity [17], were assessed using a separate model and demonstrated a statistically significant association between NTproBNP and mortality. In prognostic studies it is hard to capture how changes in management based on the value of the predictor may impact the association under study. If there is a change in management that will substantially improve prognosis, the results would lead to no association. Despite this, our study found a significant association between NTproBNP and outcomes. The use of repeated measurements somehow reduces the bias imposed by changes in management based on NTproBNP levels. Finally, our study included only CF-LVAD patients with various CF device types and patient populations that received LVAD for destination therapy, or bridge to transplantation, candidacy, decision or recovery. This increased the generalizability of the results, and our sub-analyses did not demonstrate any statistically significant association between NTproBNP and outcomes when taking these factors into consideration.

## Conclusion

Our two-centre study demonstrates that in patients with a CF-LVAD, whether used as a bridge to transplantation or destination therapy, there is a significantly increased risk in mortality and heart failure hospitalizations as NTproBNP values rise. Therefore, NTproBNP can be a useful and cost-effective clinical biomarker to predict outcomes in patients with a CF-LVAD.

## Electronic supplementary material

Below is the link to the electronic supplementary material.

Supplementary Material 1

## Data Availability

No datasets were generated or analysed during the current study.

## Abbreviations

ACEi: Angiotensin Converting Enzyme Inhibitors
ARB: Angiotensin Receptor Blockers
ARNI: Angiotensin Receptor Neprilysin Inhibitors
BMI: Body Mass Index
BNP: Brain Natriuretic Peptide
CF-LVAD: Continuous Flow Left Ventricular Assist Device
CI: Confidence Interval
DT: Destination Therapy
EPR: Electronic Patient Record
TGH: Toronto General Hospital
HR: Hazard Ratio
HTx: Heart Transplant
INTERMACS: Interagency Registry for Mechanically Assisted Circulatory Support
LVAD: Left Ventricular Assist Device
NTproBNP: N-Terminal pro Brain Natriuretic Peptide
RH: Rigshospitalet
RVAD: Right Ventricular Assist Device
SGLT2i: Sodium-glucose cotransporter 2 inhibitors
