# Supplementary material for: A two-centre study investigating the association between brain natriuretic peptides & outcomes in continuous flow left ventricular assist device recipients
Source: J Cardiothorac Surg. 2025 Apr 16;20:200. doi: 10.1186/s13019-025-03422-w (PMC12001697; doi:10.1186/s13019-025-03422-w)

**SUPPLEMENTARY FIGURE S1** – **Flow diagram of patient selection.**


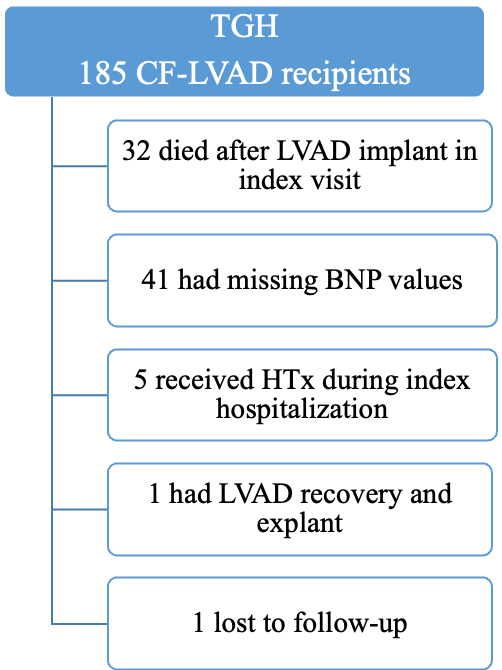

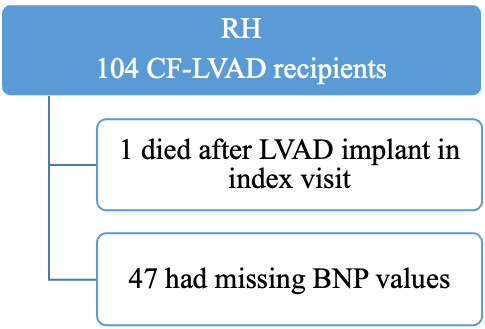

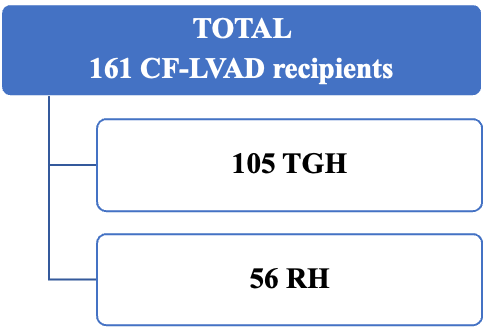


**Supplementary Table 1. Baseline Characteristics by centre.**

|  | **Overall (N=161)** | **RH (N=56)** | **TGH (N=105)** | **p-value** |
| --- | --- | --- | --- | --- |
| **Age (years)** | 53.9 [46.2, 63.8] | 54.8 [49.5, 64.7] | 53.3 [46.0, 63.0] | 0.477 |
| **Gender** |  |  |  | 0.039 |
| Male | 136 (84.5%) | 51 (92.7%) | 85 (80.2%) |  |
| Female | 25 (15.5%) | 4 (7.1%) | 21 (20.0%) |  |
| **Race** |  |  |  | <0.001 |
| Caucasian/white | 126 (78.3%) | 54 (98.2%) | 72 (67.9%) |  |
| Black | 9 (5.6%) | 0 (0%) | 9 (8.5%) |  |
| Other | 1 (0.6%) | 1 (1.9%) | 25 (23.6%) |  |
| **Body Mass Index (kg/m^2^)** | 26.5 [23.1, 29.2] | 26.6 [24.9, 28.9] | 26.4 [22.7, 29.5] | 0.799 |
| **Ischemic Cardiomyopathy** | 70 (43.5%) | 20 (35.7%) | 50 (47.6%) | 0.156 |
| **Diabetes** | 57 (35.4%) | 16 (29.1%) | 41 (38.7%) | 0.333 |
| **Smoking** |  |  |  | <0.001 |
| Never smoked | 88 (54.7%) | 22 (40.0%) | 66 (62.3%) |  |
| Currently smoking | 33 (20.5%) | 21 (38.2%) | 12 (11.3%) |  |
| Past smoker | 36 (22.4%) | 9 (16.1%) | 27 (25.5%) |  |
| **Atrial Fibrillation** | 72 (44.7%) | 36 (64.3%) | 36 (34.3%) | <0.001 |
| **COPD** | 12 (7.5%) | 3 (5.5%) | 9 (8.5%) | 0.547 |
| **Hypertension** | 68 (42.9% | 18 (32.7%) | 51 (48.1%) | 0.123 |
| **Peripheral Vascular Disease** | 12 (7.5%) | 3 (5.5%) | 9 (8.5%) | 0.749 |
| **Chronic Renal Failure** | 34 (21.1%) | 5 (9.1%) | 29 (27.4%) | 0.004 |
| **Dialysis** | 13 (8.1%) | 10 (18.2%) | 3 (2.8%) | 0.001 |
| **INTERMACS** |  |  |  | <0.001 |
| 1-2 | 23 (14.3%) | 11 (19.6%) | 12 (11.4%) |  |
| 3-4 | 115 (71.4%) | 27 (48.2%) | 88 (83.8%) |  |
| 5-7 | 17 (10.6%) | 15 (26.8%) | 2 (1.9%) |  |
| **Hemoglobin (g/L)** | 11.9 [9.8, 13.3] | 12.4 [ 11.4, 14.0] | 11.3 [9.5, 12.8] | 0.001 |
| **Creatinine (μmol/L)** | 114.0 [90.1, 151.9] | 138.0 [95.5, 167.0] | 121.0 [86.7, 141.4] | 0.06 |
| **LVAD Type** |  |  |  |  |
| HeartMate II | 77 (47.8%) | 34 (61.8%) | 43 (40.6%) | <0.001 |
| HeartMate III | 46 (28.6%) | 21 (38.2%) | 25 (23.6%) |  |
| HeartWare | 37 (23.0%) | 0 (0%) | 37 (34.9%) |  |
| Other | 1 (0.6%) | 0 (0%) | 1. (0.9%) |  |

*TGH: Toronto General Hospital; RH: Rigshospitalet.*

**Supplementary Table 2. Pre-LVAD transthoracic echocardiogram and right heart catheterization.**

| **Parameters** | **TGH** | **RH** | **Overall** | **p-value** |
| --- | --- | --- | --- | --- |
| **Transthoracic echocardiogram** | | | | |
| LVEF | 19.0 [15.0, 21.0] | 10.0 [10.0, 15.0] | 10.0 [10.0, 15.0] | p<0.005 |
| LVEDD | 6.70 [6.00, 7.30] | 7.1 [6.4, 7.8] | 7.00 [6.4, 7.7] | 0.14 |
| LA Volume | 112.2 [89.2, 142.7] | 122.3 [98.7, 169.0] | 110.7 [91.6, 169.0] | 0.40 |
| LAVi | 59.6 [53.3, 77.0] | 59.7 [47.1, 74.7] | 59.2 [47.1, 74.7] | 0.40 |
| RVEF Grading | 2.00 [1.0, 2.0] | 2.0 [0.0, 2.0] | 1.0 [0.0, 2.0] | 0.74 |
| RVd | 4.70 [4.2, 5.10] | 3.4 [3.2, 3.7] | 3.9 [3.4, 4.1] | 0.05 |
| RVSP | 52.5 [42.3, 61.0] | 50.0 [47.5, 52.5] | 45.0 [40.0, 55.0] | 0.77 |
| TAPSE | 1.9 [1.4, 13.5] | 1.5 [1.2, 1.8] | 1.5 [1.2, 1.8] | p<0.005 |
| **Right heart catheterization** | | | | |
| CO | 3.8 [3.1, 5.0] | 3.9 [3.4, 4.7] | 4.0 [3.4, 4.9] | 0.72 |
| mRAP | 8.0 [4.0, 11.0] | 14.0 [10.0, 18.0] | 14.0 [10.8, 18.0] | p<0.005 |
| RVSP2 | 51.0 [38.5, 68.0] | 53.5 [42.0, 60.5] | 52.0 [42.0, 60.0] | 0.45 |
| RVDP | 6.0 [3.0, 10.0] | 12.0 [8.8, 16.0] | 12.0 [8.5, 17.0] | p<0.005 |
| PASP | 46.5 [38.5, 63.0] | 53.5 [42.5, 62.0] | 50.0 [42.0, 61.0] | 0.25 |
| PADP | 23.5 [18.3, 27.8] | 30.0 [24.0, 34.0] | 28.0 [24.0, 34.0] | p<0.005 |
| mPAP | 32.0 [27.0, 41.0] | 36.7 [32.1, 42.8] | 36.3 [32.0, 42.7] | 0.02 |
| mPAWP | 22.0 [17.0, 27.0] | 27.0 [24.0, 30.0] | 27.0 [25.0, 30.0] | p<0.005 |
| TPG | 12.0 [8.0, 17.0] | 18.5 [16.8, 20.3] | 15.0 [15.0, 22.0] | 0.35 |
| PVR | 2.6 [1.0, 3.6] | 2.6 [1.7, 3.4] | 2.5 [1.5, 3.2] | 0.56 |

*LVEF: Left Ventricular Ejection Fraction; LVEDD: Left Ventricular End-Diastolic Diameter; LA Volume: Left Atrial Volume; LAVi: Left Atrial Volume Index; RVEF Grading: Right Ventricular Ejection Fraction Grading; RVd: Right Ventricular Diameter; RVSP: Right Ventricular Systolic Pressure; TAPSE: Tricuspid Annular Plane Systolic Excursion; CO: Cardiac Output; mRAP: Mean Right Atrial Pressure; RVSP2: Alternative Right Ventricular Systolic Pressure Measurement; RVDP: Right Ventricular Diastolic Pressure; PASP: Pulmonary Artery Systolic Pressure; PADP: Pulmonary Artery Diastolic Pressure; mPAP: Mean Pulmonary Artery Pressure; mPAWP: Mean Pulmonary Artery Wedge Pressure; TPG: Transpulmonary Gradient; PVR: Pulmonary Vascular Resistance.*

**Supplementary Table 3. Comparison of medical therapy and patient outcomes.**

| **Medication** | **Alive (N, %)** | **Dead (N, %)** | **P-value** |
| --- | --- | --- | --- |
| Beta-Blocker | 53 (63.1%) | 10 (50.0%) | 0.41 |
| Angiotensin-Converting Enzyme Inhibitor | 40 (47.6%) | 9 (45.0%) | 1 |
| Angiotensin II Receptor Blocker | 4 (4.8%) | 2 (10.0%) | 0.71 |
| Angiotensin Receptor-Neprilysin Inhibitor | 0 (0.0%) | 0 (0.0%) |  |
| Mineralocorticoid Receptor Antagonist | 0 (0.0%) | 0 (0.0%) | 0.92 |
| Sodium-Glucose Cotransporter-2 Inhibitor | 0 (0.0%) | 0 (0.0%) |  |
| Hydralazine | 26 (31.0%) | 4 (20.0%) | 0.49 |
| Isosorbide Dinitrate | 6 (7.1%) | 2 (10.0%) | 1 |
| Sildenafil | 27 (32.1%) | 6 (30.0%) | 1 |
| Furosemide (Lasix) | 72 (85.7%) | 18 (90.0%) | 0.89 |

**Supplementary Table 4. Other covariates in the multivariable Cox Regression Model for the association between NTproBNP and mortality.**

|  | Hazard ratio | 95% Confidence Interval |
| --- | --- | --- |
| Age | 1.0 | 0.9 – 1.0 |
| Gender | 0.7 | 0.3 – 1.4 |
| Diabetes | 1.2 | 0.6 – 2.2 |
| Ischemic Cardiomyopathy | 0.9 | 0.5 – 1.8 |

**Supplementary Figure S2 Univariable Cox Regression Model for Heart Transplantation.**

Association between NTproBNP and heart transplant analyzed using univariable Cox regression model. NTproBNP was entered as a time-dependent covariate modeling multiple measures per patient and using cubic spline, with death as the competing event.

**
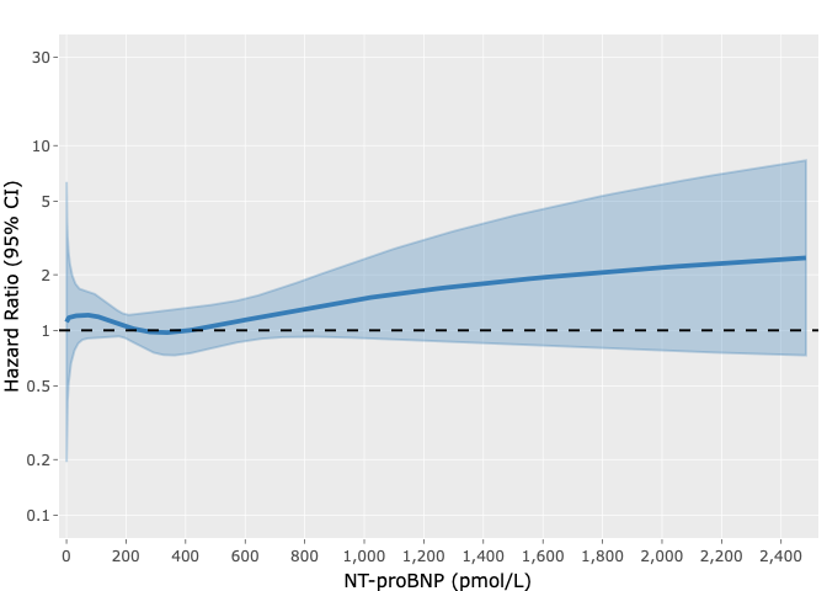
**

**Supplementary Table 5. Other covariates in the multivariable Cox Regression Model for the association between NTproBNP and heart transplantation.**

|  | Hazard ratio | 95% Confidence Interval |
| --- | --- | --- |
| Age | 0.9 | 0.9 – 1.0 |
| Gender | 1.6 | 0.8 – 3.1 |
| Diabetes | 0.9 | 0.5 – 1.5 |
| Ischemic Cardiomyopathy | 0.8 | 0.5 – 1.3 |

**Supplementary Table 6. Other covariates in the multivariable cause-specific cox regression model for the association between NTproBNP and Heart Failure Hospitalizations.**

|  | Hazard ratio | 95% Confidence Interval |
| --- | --- | --- |
| Age | 1.0 | 0.9 – 1.1 |
| Gender | 0.9 | 0.4 – 2.2 |
| Diabetes | 1.3 | 0.7 – 2.4 |
| Ischemic Cardiomyopathy | 0.4 | 0.2 – 0.8 |

**Supplementary Table 7. Other covariates in the multivariable Cox Regression Model incorporating renal function and BMI to determine the association between NTproBNP and Heart Transplantation. The association between NT-pro BNP and Heart Transplantation is described in Figure S3.**

|  | Hazard ratio | 95% Confidence Interval |
| --- | --- | --- |
| Creatinine | 0.9 | 0.9 – 1.0 |
| Dialysis | 0.4 | 0.1 – 1.5 |
| BMI (kg/m^2^) | 1.0 | 0.9 – 1.1 |

**Supplementary Figure S3. Multivariable Cox Regression Model for Heart Transplantation accounting for renal function and BMI.**

Association between NTproBNP and heart transplant analyzed using multivariable Cox regression model. NTproBNP was entered as a time-dependent covariate modeling multiple measures per patient and using cubic spline, with death as the competing event. The multivariable analysis was adjusted for creatinine, dialysis and BMI.

**
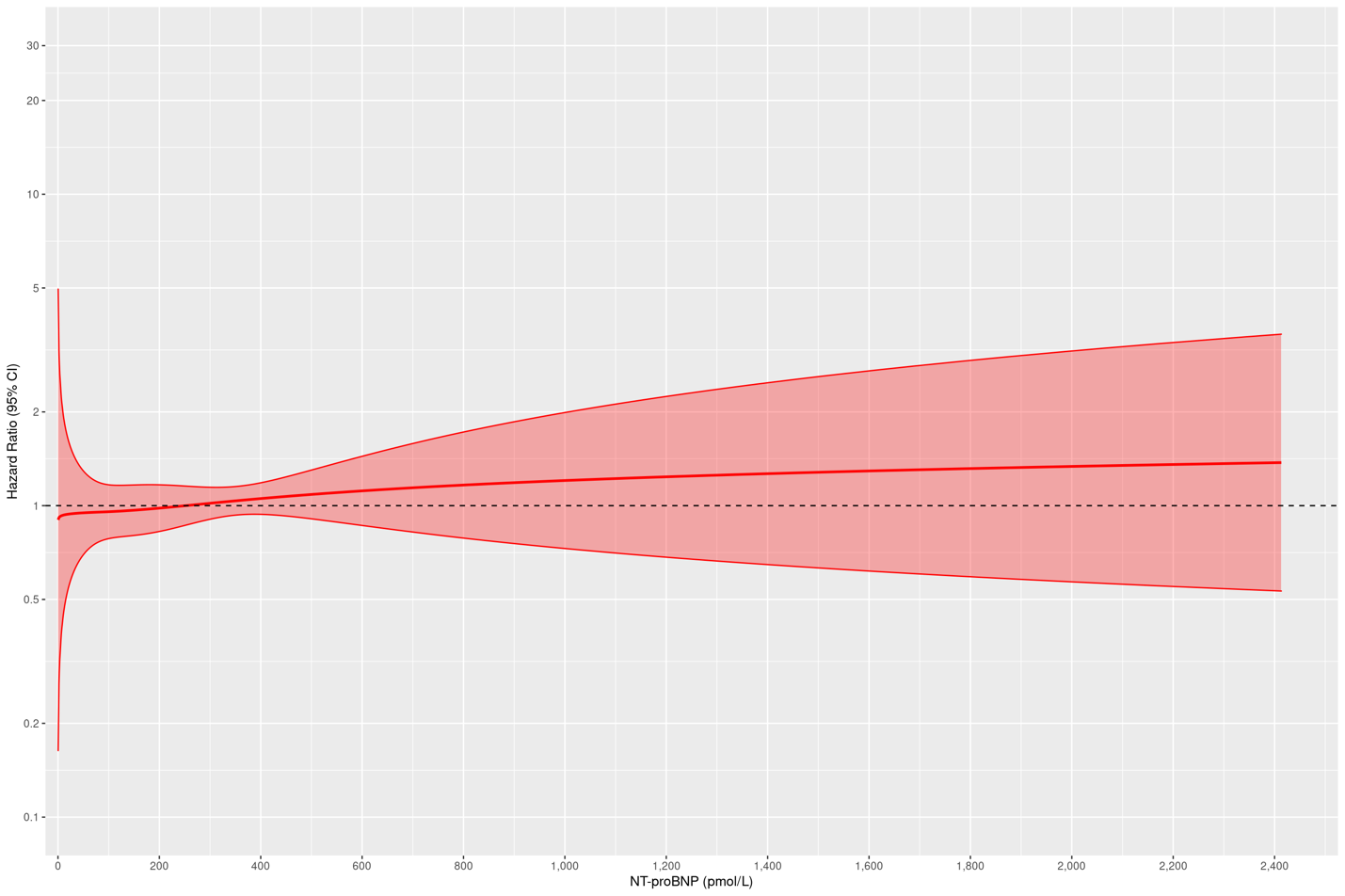
**

**Supplementary Table 8. Other covariates in the multivariable Cox Regression Model incorporating renal function and BMI to determine the association between NTproBNP and mortality. The association between NT-pro BNP and Mortality is described in Figure S4.**

|  | Hazard ratio | 95% Confidence Interval |
| --- | --- | --- |
| Creatinine | 1.0 | 0.9 – 1.0 |
| Dialysis | 0.3 | 0.1 – 1.0 |
| BMI (kg/m^2^) | 1.0 | 0.9 – 1.1 |

**Supplementary Figure S4. Multivariable Cox Regression Model for Mortality accounting for renal function and BMI.**

Association between NTproBNP and mortality analyzed using multi-variable Cox regression model. NTproBNP was entered as a time-dependent covariate modeling multiple measures per patient and using cubic spline, with heart transplantation as the competing event. The multivariable analysis was adjusted for creatinine, dialysis and BMI.


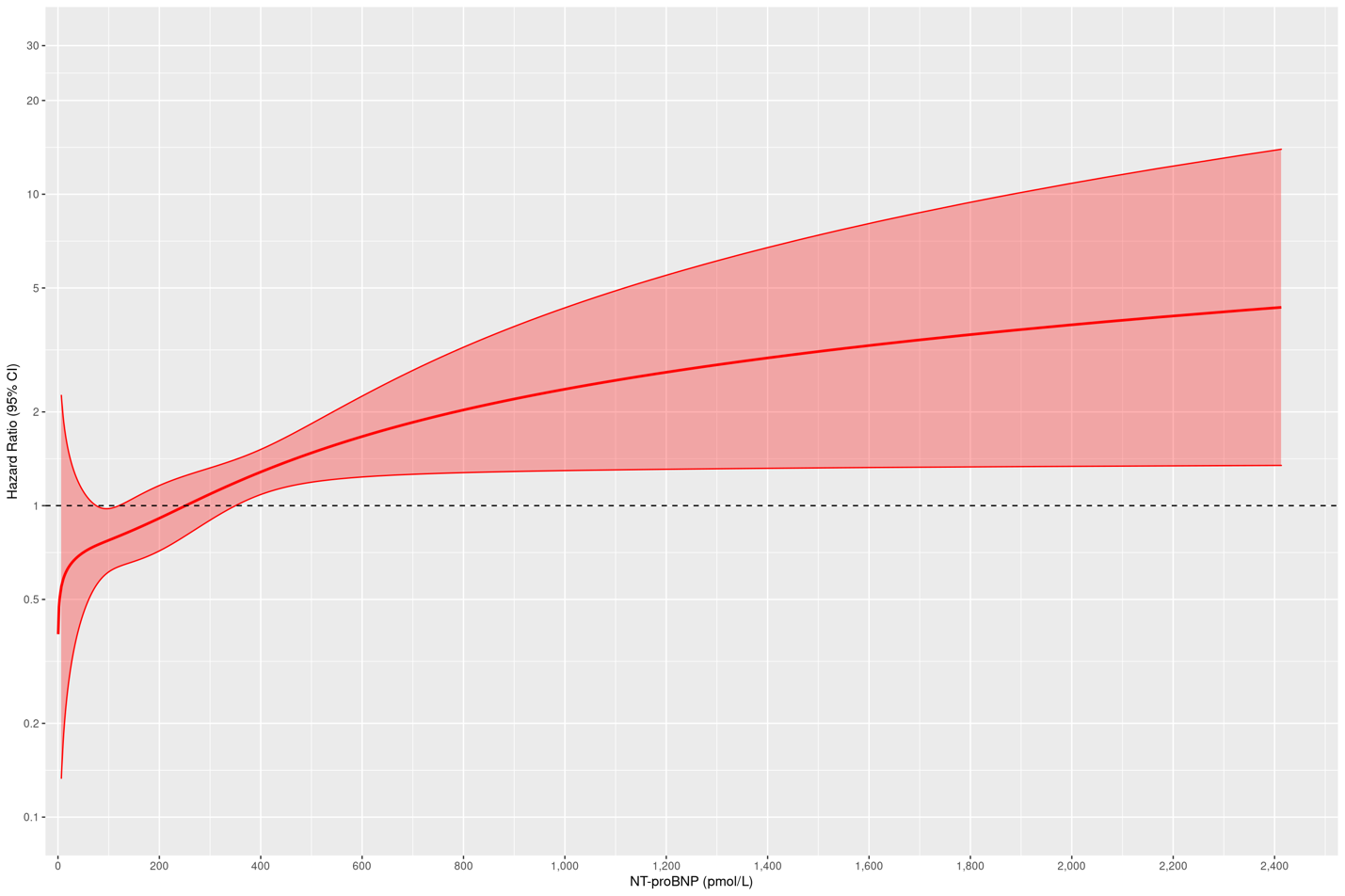


**Supplementary Table 9. Other covariates in the multivariable Cox Regression Model for Mortality accounting for centre and intention of LVAD therapy. The association between NT-pro BNP and mortality is described in Figure S5.**

|  | Hazard ratio | 95% Confidence Interval |
| --- | --- | --- |
| Centre – TGH vs. RH | 1.05 | 0.53 – 2.11 |
| Intention of LVAD – DT vs. BTT | 1.33 | 0.56 – 3.15 |
| Intention of LVAD – Other vs. BTT | 1.26 | 0.60 – 2.66 |

*TGH: Toronto General Hospital; RH: Rigshospitalet; LVAD: Left Ventricular Assist Device; DT: Destination Therapy; BTT: Bridge to Transplantation; Other: Grouped LVAD patients who were bridged to decision, candidacy and recovery.*

**Supplementary Figure S5. Multivariable Cox Regression Model for Mortality accounting for centre and intention of LVAD therapy.**

Association between NTproBNP and mortality analyzed using multi-variable Cox regression model. NTproBNP was entered as a time-dependent covariate modeling multiple measures per patient and using cubic spline, with heart transplantation as the competing event. The multivariable analysis was adjusted for the intention of LVAD therapy and center.


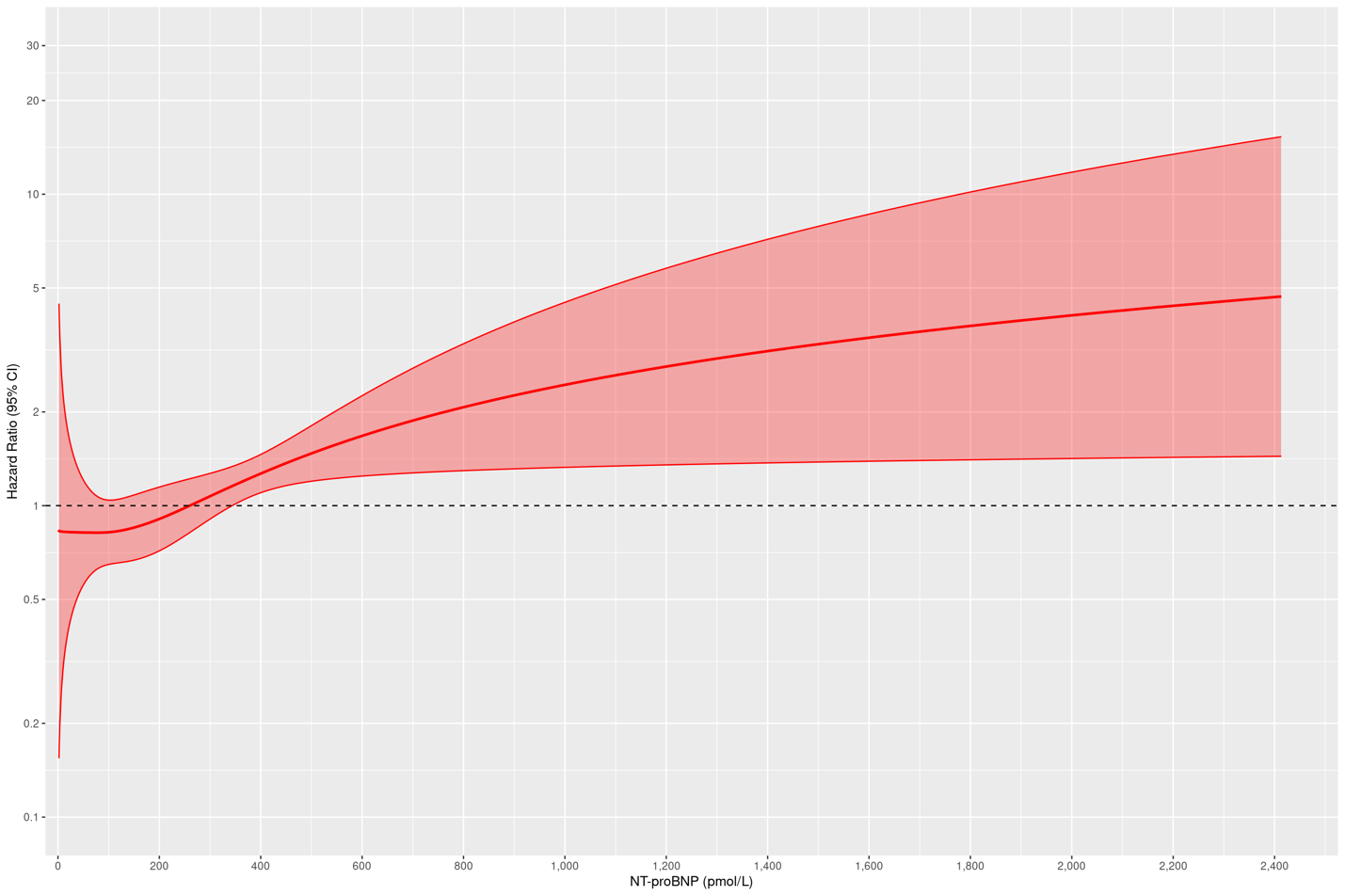


**Supplementary Table 10. Other covariates in the multivariable Cox Regression Model for Heart Transplantation accounting for centre and intention of LVAD therapy. The association between NT-pro BNP and heart transplantation is described in Figure S6.**

|  | Hazard ratio | 95% Confidence Interval |
| --- | --- | --- |
| Centre – TGH vs. RH | 1.08 | 0.65 – 1.79 |
| Intention of LVAD – DT vs. BTT | 0.03 | 0.004 – 0.211 |
| Intention of LVAD – Other vs. BTT | 0.64 | 0.40 – 1.02 |

*TGH: Toronto General Hospital; RH: Rigshospitalet; LVAD: Left Ventricular Assist Device; DT: Destination Therapy; BTT: Bridge to Transplantation; Other: Grouped LVAD patients who were bridged to decision, candidacy and recovery.*

**Supplementary Figure S6. Multivariable Cox Regression Model for Mortality accounting for centre and intention of LVAD therapy.**

Association between NTproBNP and heart transplantation analyzed using multi-variable Cox regression model. NTproBNP was entered as a time-dependent covariate modeling multiple measures per patient and using cubic spline, with mortality as the competing event. The multivariable analysis was adjusted for the intention of LVAD therapy and centre.


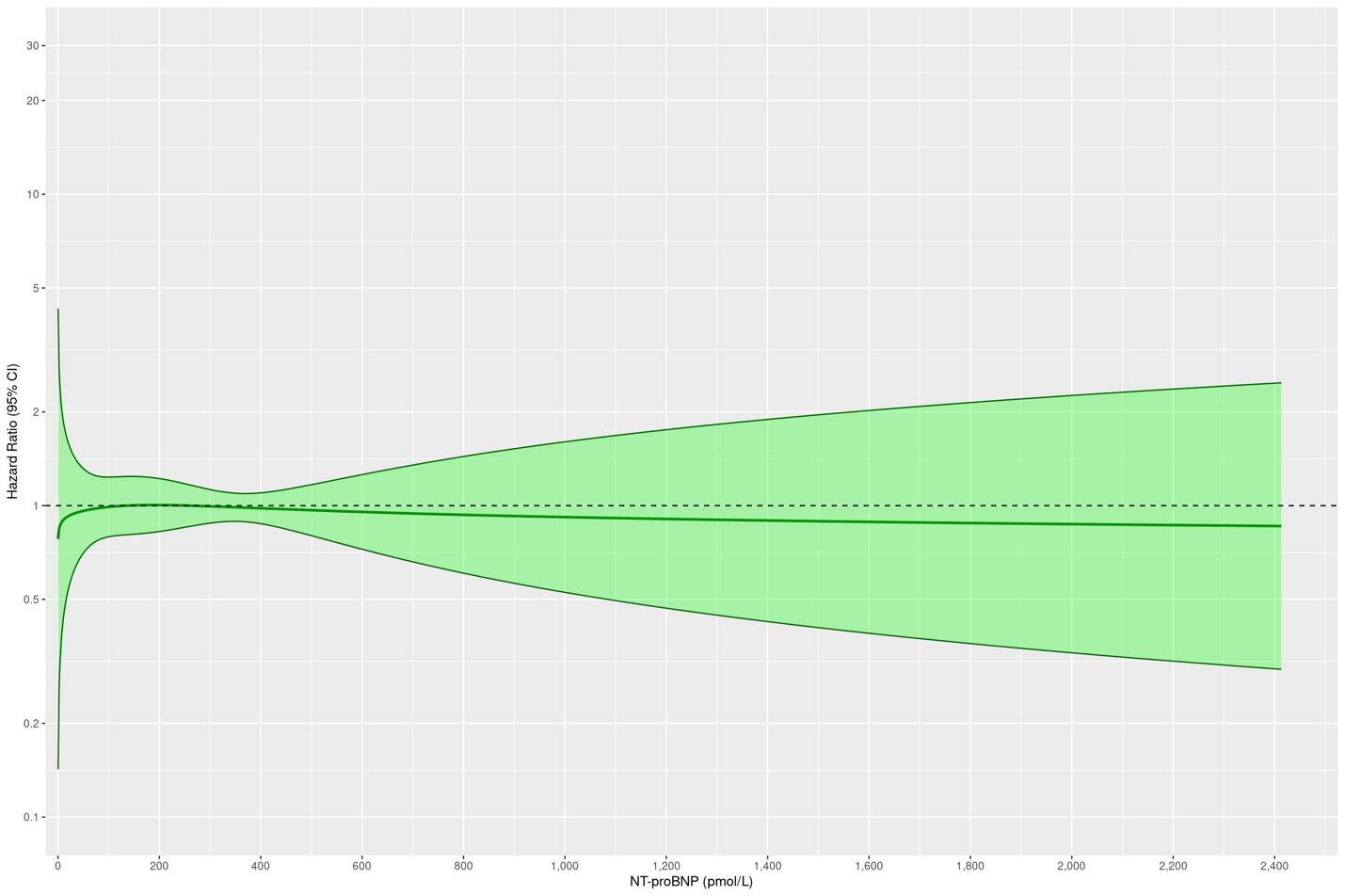

Supplement: Supplementary file 1 — Supplementary Material 1 [file 13019_2025_3422_MOESM1_ESM.docx]
